# Motion artifacts removal and evaluation techniques for functional near-infrared spectroscopy signals: A review

**DOI:** 10.3389/fnins.2022.878750

**Published:** 2022-10-03

**Authors:** Ruisen Huang, Keum-Shik Hong, Dalin Yang, Guanghao Huang

**Affiliations:** ^1^School of Mechanical Engineering, Pusan National University, Busan, South Korea; ^2^Department of Cogno-Mechatronics Engineering, Pusan National University, Busan, South Korea; ^3^Mallinckrodt Institute of Radiology, Washington University School of Medicine, St. Louis, MO, United States; ^4^Institute for Future, School of Automation, Qingdao University, Qingdao, China

**Keywords:** functional near-infrared spectroscopy, motion artifacts removal, hemodynamic response, filtering techniques, noise suppression, signal-to-noise ratio

## Abstract

With the emergence of an increasing number of functional near-infrared spectroscopy (fNIRS) devices, the significant deterioration in measurement caused by motion artifacts has become an essential research topic for fNIRS applications. However, a high requirement for mathematics and programming limits the number of related researches. Therefore, here we provide the first comprehensive review for motion artifact removal in fNIRS aiming to (i) summarize the latest achievements, (ii) present the significant solutions and evaluation metrics from the perspective of application and reproduction, and (iii) predict future topics in the field. The present review synthesizes information from fifty-one journal articles (screened according to three criteria). Three hardware-based solutions and nine algorithmic solutions are summarized, and their application requirements (compatible signal types, the availability for online applications, and limitations) and extensions are discussed. Five metrics for noise suppression and two metrics for signal distortion were synthesized to evaluate the motion artifact removal methods. Moreover, we highlight three deficiencies in the existing research: (i) The balance between the use of auxiliary hardware and that of an algorithmic solution is not clarified; (ii) few studies mention the filtering delay of the solutions, and (iii) the robustness and stability of the solution under extreme application conditions are not discussed.

## Introduction

Functional near-infrared spectroscopy (fNIRS) is a non-invasive brain imaging technique that uses near-infrared light (typically of wavelengths between 650 and 1,000 nm) to monitor hemodynamics changes in the cortical layer. Compared to electroencephalography (EEG), fNIRS enables to measure brain-activity related hemodynamics in terms of cerebral oxygenation and is less susceptible to electric noises (Huppert et al., 2009; Tak and Ye, 2014; Naseer and Hong, 2015; Chiarelli et al., 2017; Afkhami et al., 2019; Ghafoor et al., 2019; Khan et al., 2021). In addition, fNIRS can be integrated into a portable, wearable, and ergonomic device at low costs and operational expenses, making it a superior candidate for a user-friendly brain-computer interface system compared to other modalities, such as functional magnetic resonance imaging (fMRI) and magnetoencephalography (MEG) (Hu et al., 2010; Piper et al., 2014; Scholkmann et al., 2014a; Pinti et al., 2015; Wyser et al., 2017; Zhao and Cooper, 2018; Hong and Zafar, 2018; Zhao H. B. et al., 2020, 2021; Ghafoor et al., 2021; Huang and Hong, 2021).

Fantini et al. (1999) reported that the artifacts caused by subjects’ movements, specifically motion artifacts (MAs), can significantly influence the quality of the recorded optical signals of fNIRS. Some studies claimed that MAs reduce the signal-to-noise ratio (SNR) of fNIRS signals (Izzetoglu et al., 2005; Izzetoglu et al., 2010). Researchers have also verified that using MA removal techniques can ameliorate classification accuracy in cognition experiments (Zhou et al., 2021). Therefore, the issue concerning the causes, characteristics, and rejection methods of MAs in fNIRS signals is a crucial topic in fNIRS studies (Safaie et al., 2013; Piper et al., 2014).

During the early days, researchers skipped the analysis or discarded the data set when the measured signals were significantly corrupted by motion artifacts (Bartocci et al., 2000; Jasdzewski et al., 2003; Akgul et al., 2005; Khan and Hong, 2015; Nguyen and Hong, 2016; Zafar and Hong, 2017). Schroeter et al. (2003) removed the outliers manually. Moosmann et al. (2003) eliminated the disturbances by immobilizing the subjects’ heads with a vacuum pad. Subsequently, a moving average was used (Kameyama et al., 2004; Lee et al., 2007). Channel rejection is another common method in early studies (Wilcox et al., 2005; Blasi et al., 2010). Attempts were made to remove MAs using an improved optical model. Nevertheless, the performances of the aforementioned solutions were not sufficient (Scholkmann et al., 2014b). Nowadays, a correction for motion artifacts has become a common consensus: Some processed the signals in two stages: artifact identifications and artifact corrections in the existing methods (Scholkmann et al., 2010; Virtanen et al., 2011; Yucel et al., 2014a), or minimized a user-defined cost function (Kim et al., 2011), or proposed a new model to compensate the artifacts (Izzetoglu et al., 2010; Yamada et al., 2015).

The existing literature indicates that the movements that cause MAs are diverse. Several studies have reported that the movements of subjects’ heads (including nodding, shaking, tilting, etc.) could result in MAs in fNIRS measurements (Izzetoglu et al., 2005; Radhakrishnan et al., 2009; Robertson et al., 2010; Kim et al., 2011; Cui et al., 2015). Some researchers further discovered that facial muscle movement, including raising eyebrows, can lead to MAs (Izzetoglu et al., 2005; Robertson et al., 2010; Yucel et al., 2014b; Zhou et al., 2021). In addition, body movements, including the movement of the upper and lower limbs, degrade the fNIRS signals by causing head movements or by the inertia of the device (Yucel et al., 2014a; Rea et al., 2014; Abtahi et al., 2017; Vitorio et al., 2017; Khan et al., 2018; Siddiquee et al., 2020; Dybvik and Steinert, 2021). Vinette et al. (2015) monitored five epilepsy patients for a long period. Their data showed that MAs existed when the subjects were talking, eating, or drinking. These behaviors involve jaw movements. Novi et al. (2020) found that jaw movements could lead to two different motion artifacts. The direct cause of MAs is imperfect contact between the optodes and the scalp, including displacement, non-orthogonal contact, and oscillation of the optodes (Yamada et al., 2015; Nishiyori, 2016).

**FIGURE 1:**
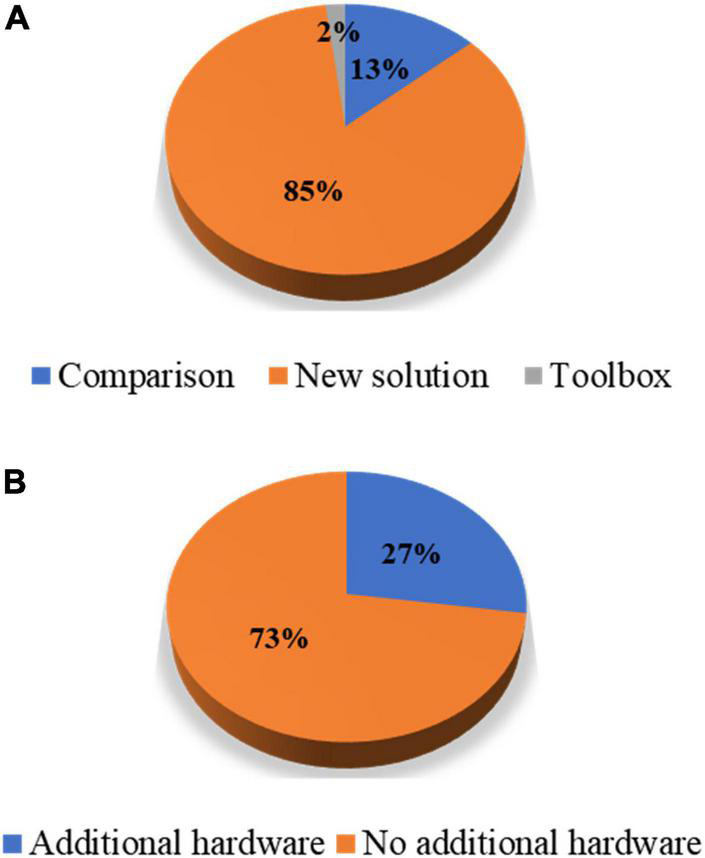
Percentage partitions: **(A)** Article types of the selected papers and **(B)** hardware-based solutions against algorithmic solutions among the papers proposing new solutions.

In this study, the authors reviewed journal articles concerning MA removal techniques in the Web of Science database. The keywords and numbers of journal papers are listed in Table 1. To narrow down our review scope, we list all journal articles found in the database. Subsequently, all the overlapping papers and irrelevant articles were removed by examining their context, which yielded 89 papers. Next, we proceeded to select journal papers that satisfied at least one of the following criteria: (i) The paper proposes a novel MA removal technique; (ii) the paper presents a quantitative comparison of several MA removal techniques; and (iii) the paper introduces a toolbox for MA removal. Eventually, 55 papers were selected from the literature. Forty-three papers presented a new solution to suppress MAs, seven papers compared the performance of the existing methods, and one study introduced a toolbox. Figure 1 shows the partitioning of different types of papers in the selection process. Among the 47 new solutions, twelve solutions added auxiliary hardware. A list of selected studies and their categories is presented in Table 2. Since research on MA removal techniques requires a solid foundation in mathematics and programming, it is difficult for new scholars to assimilate the existing solutions in their studies. Moreover, some solutions were described in the text rather than using equations, making it difficult for other researchers to reproduce the reported methodologies. Therefore, this study aims to (i) provide a general view of the latest achievements in MA removal studies, (ii) briefly introduce several significant solutions from the view point of application and reproduction by using equations, and (iii) discuss future topics in the field.

**TABLE 1:** The number of journal papers (1990∼2022) obtained from the Web of Science database by combining different keywords.

Keywords	Paper number
fNIRS + motion artifact	90
NIRS + motion artifact	61
NIRS + motion correction	18
fNIRS + motion correction	27
Functional near-infrared spectroscopy + motion correction	32
Near-infrared spectroscopy + motion correction	51
Near-infrared spectroscopy + motion artifact	150
Functional near-infrared spectroscopy + motion artifact	109

**TABLE 2:** List of selected papers, article types, and information on additional hardware.

Paper	Type	Additional hardware
Izzetoglu et al. (2005)	New solution	
Zhang et al. (2005)	New solution	
Lee et al. (2007)	New solution	
Blasi et al. (2010)	New solution	Accelerometer
Cui et al. (2010)	New solution	
Izzetoglu et al. (2010)	New solution	
Robertson et al. (2010)	Comparison	
Scholkmann et al. (2010)	New solution	
Kim et al. (2011)	New solution	Accelerometer
Virtanen et al. (2011)	New solution	Accelerometer
Cooper et al. (2012)	Comparison	
Molavi and Dumont (2012)	New solution	
Amian and Setarehdan (2013)	New solution	
Barker et al. (2013)	New solution	
Santosa et al. (2013)	New solution	
Sweeney et al. (2013)	New solution	
Takahashi et al. (2013)	New solution	3D motion capture system
Brigadoi et al. (2014)	Comparison	
Selesnick et al. (2014)	New solution	
Yucel et al. (2014b)	New solution	Collodion-fixed prism-based optical fibers
Yucel et al. (2014a)	New solution	
Chiarelli et al. (2015)	New solution	
Hu et al. (2015)	Comparison	
Metz et al. (2015)	New solution	Accelerometer
Yamada et al. (2015)	New solution	Linearly polarized light sources, an orthogonally polarized analyzer
Barker et al. (2016)	New solution	
Gu et al. (2016)	New solution	
Guerrero-Mosquera et al. (2016)	New solution	
Islam et al. (2017)	New solution	Accelerometer
Janani and Sasikala (2017)	Comparison	
Santosa et al. (2017)	New solution	
Behrendt et al. (2018)	Comparison	
Dong and Jeong (2018)	New solution	
Jahani et al. (2018)	New solution	
Lee et al. (2018)	New solution	
Nguyen et al. (2018)	New solution	
Reyes et al. (2018)	Comparison	
Shukla et al. (2018)	New solution	
Siddiquee et al. (2018)	New solution	Inertia measurement unit (IMU)/accelerometer, gyroscope, magnetometer
Sutoko et al. (2018)	New solution	
Di Lorenzo et al. (2019)	Comparison	
Fishburn et al. (2019)	New solution	
Seghouane and Ferrari (2019)	New solution	
von Luhmann et al. (2019)	New solution	Accelerometer
Arunkumar and Bhaskar (2020)	New solution	
Gao et al. (2020)	New solution	
Sherafati et al. (2020)	New solution	
Zhao J. et al. (2020)	New solution	
Gemignani and Gervain (2021)	Comparison	
Lacerenza et al. (2021)	New solution	TD-fNIRS
Perpetuini et al. (2021)	New solution	Camera
Zhao Y. et al. (2021)	Toolbox	
Zhou et al. (2021)	New solution	
Gao L. et al. (2022)	New solution	
Kim et al. (2022)	New solution	
Huang et al. (2022)	New solution	
Gao Y. et al. (2022)	New solution	

This study is divided into five sections. The “Introduction” section presents the causes and significance of MA issues in fNIRS. In addition, this section specifies the objectives of this study and provides a quantitative summary of the existing literature on the topics. The section “Additional hardware-based techniques” summarizes the existing hardware-based solutions. The section “Signal processing-based techniques” discusses the algorithmic solutions. The section “Evaluation metrics” briefly introduces the definitions of some metrics to evaluate the performance of MA removal techniques. The final section “Conclusions and outlook” concludes this study and discusses potential issues concerning MA removal. We will use the compact notation provided in Table 3 for the remainder of the paper.

**TABLE 3:** Definitions of variables, parameters, and their values.

Variables/Parameters	Definitions
*z*(*n*)	Measured signals
*x*(*n*)	Motionless signals
*v*(*n*)	Motion artifacts
$\hat{x}$(*n*)	Estimated motionless functional near-infrared spectroscopy (fNIRS) signals
$\hat{v}$(*n*)	Estimated motion artifacts
*a*(*n*)	Accelerometer output
Δ*t*	Sampling interval
∨	OR operation
*flag*_*ME*_	Flag for motion events
*T*_*m*_	Timing of a motion event starts
*T*_*before*_	5 s before *T*_*m*_
*T*_*after*_	5 s after *T*_*m*_
*Avg*_*before*_	Signals’ amplitudes before *T*_*m*_
*Avg*_*after*_	Signals’ amplitudes after *T*_*m*_
*flag*_*BS*_	Flag identifying the baseline shifts in motion events
std(⋅)	Standard deviation of its input
*flag*_*crr*_	Flag for correction
∧	AND operation
*N*_*ch*_(⋅)	Number of channels satisfying a given condition
*N*_*wv*_(⋅)	Number of wavelengths satisfying a given input
*G*	Gravity acceleration approximating 9.81 m/s^2^
*r*_*s*_(*n*)	Light transmittances of the source-scalp gap
*r*_*d*_(*n*)	Light transmittances of the detector-scalp gap
*N*	Time instance
*I*_0_	Light intensity emitted by the source
*I*_1_(*n*)	Light intensity reflected by hair
*I*_2_(*n*)	Light intensity scattered by head tissue
*DPF*	Differential pathlength factor
*D*	Source-detector distance
Δμ	Absorption coefficients change in the gray matter
*g*(*n*)	Wiener filter
*p*_*x*_(*w*)	Power spectral densities (PSD) of the actual fNIRS signals
*p*_*v*_(*w*)	PSD of the motion artifacts
*a*_*i*_	Parameters of the AR model
ϕ(*n*)	Composed of *p* motion artifact-free fNIRS signals
ω*_*n*_*	Zero-mean noises in the AR model
*Q*	Error covariance matrix
ν*_*n*_*	Measurement noise with an error covariance matrix *R*
*W*	Samples of moving time window
*msd*(*n*)	Moving standard deviation
*ξ*(*n*)	Corresponding samples
*z*_*MA*_(*n*)	Motion artifacts segments
*z*_*NC*_(*n*)	Non-corrupted segments
*z*_*diff*_(*n*)	Difference between *z*_*MA*_(*n*) and its spline interpolation fitting
*i*_0_	Coarsest scale
Φ(*n*)	Mother scaling function
Ψ(*n*)	Mother wavelet function
*N*	Number of samples in the signals
NCDF(⋅)	Normal cumulative distribution function
σ_*HbO*_	Standard deviation of *z*_*HbO*_
σ_*HbR*_	Standard deviation of *z*_*HbR*_
*W*_*L*_	Large-window size
*W*_*S*_	Small-window size
*N*_*ch*_	Number of channels
*Id*	An identity matrix
ν_1_ *and* ν*_2_*	Two types of motion artifacts
Ω	Gaussian white noise
[⋅]*_*i, j*_*	Element located at the *i*th row and the *j*th column
[⋅]*_i_*	*i*th element of the vector
*W*_1_	Window size of the first median filter
*W*_2_	Window size of the second median filter
Var (⋅)	Variance of the variable in the parentheses
σ_*dur*_	Standard deviation of the filtered fNIRS signals during stimulus
σ_*pre*_	Standard deviation of the filtered fNIRS signals before stimulus
∈	Small nonnegative constant
*A*_*m*_	Set of time segments where motion artifacts (Mas) occur
*N*_*trl*_	Number of trials in the signals
*N*_*sub*_	Number of subjects
ΔCC	Difference of correlation coefficient

## Additional hardware-based techniques

Among the eight-nine selected papers, 17 discussed solutions using additional hardware, while 11 studies discussed accelerometer-related methods. Other auxiliary hardware includes a headpost cemented to the skull, a three-dimensional (3D) motion capture system, collodion-fixed prism-based optical fibers, an inertia measurement unit (IMU), a gyroscope, a magnetometer, and a camera. This section presents two solutions using accelerometers and one using linearly polarized light.

### Accelerometer

Accelerometer-based methods include adaptive filtering, active noise cancelation (ANC) (Kim et al., 2011), accelerometer-based motion artifact removal (ABAMAR) (Virtanen et al., 2011), acceleration-based movement artifact reduction algorithm (ABMARA) (Metz et al., 2015), multi-stage cascaded adaptive filtering (Islam et al., 2017), blind source separation, accelerometer-based artifact rejection, and detection (BLISSA2RD) (von Luhmann et al., 2019). The introduction of the accelerometer improves the feasibility of real-time rejection of MAs.

#### Active noise cancelation

The method assumes that the measured signals, *z*(*n*), are the sum of motionless signals, *x*(*n*), and MAs, *v*(*n*) (Kim et al., 2011). The objective of the solution is to minimize the power of the recovered signals, that is,


(1) $$\min\left(\text{E}\left(\hat{x}{\left(n\right)}^{2}\right)\right)=\min\left(\text{E}\left({\left(x\left(n\right)+v\left(n\right)-\hat{v}\left(n\right)\right)}^{2}\right)\right)$$

$$\;=\min(\text{E}\left(x{\left(n\right)}^{2}\right)+2\text{E}\left(x\left(n\right)v\left(n\right)\right)$$

$$\;-2\text{E}\left(x\left(n\right)\hat{v}\left(n\right)\right)+\text{E}\left({\left(v\left(n\right)-\hat{v}\left(n\right)\right)}^{2}\right)).$$

where *E*(⋅) denotes the expectation function, and the hat over *x* and *v* denotes the estimation of motionless fNIRS signals and MAs. Ideally, *x* is uncorrelated to either *v* or the estimate of *v*. Therefore, the two cross-terms on the right-hand side are equal to zero implying that the objective is equivalent to minimizing the square difference between the MAs and the estimated MAs. Moreover, because the actual MAs are highly correlated to the accelerometer output, *a*(*n*), but unknown to users, *v*(*n*) is replaced by *a*(*n*) in the application. Subsequently, the final objective of signal processing is to minimize the square difference between *a*(*n*) and the estimated MAs, that is,


(2) $$\min\left(\text{E}\left({\left(a\left(n\right)-\hat{v}\left(n\right)\right)}^{2}\right)\right)$$

**FIGURE 2:**
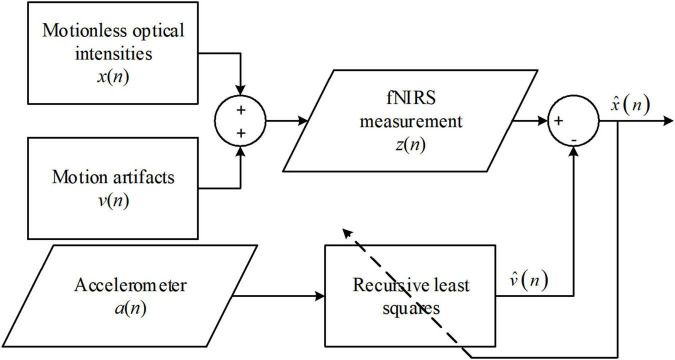
Procedures of active noise cancellation (ANC) algorithm.

The estimate of *v*(*n*) can be obtained using the difference between *z*(*n*) and the estimate of *x*(*n*). The estimate of *v*(*n*) can then be computed in real time using a recursive least-squares filter. The procedure for the solution is graphically presented in Figure 2. The ANC solution was applied to optical intensities in real time. Whether this method was applied to optical densities or concentration changes is not clear. Another issue in the approach of Kim et al. (2011) is that the performance was visually evaluated. Therefore, a quantitative evaluation of ANC in the sense of both noise suppression and signal distortion is needed.

#### Accelerometer-based motion artifact removal algorithm

The ABAMAR method is an offline analysis method for MA removal, where accelerometer outputs are used for MA detection, and the MA removal process is based on the measured fNIRS signals (Virtanen et al., 2011). Accordingly, we first define two Boolean functions as follows.


(3) $$f_{1}\left(x\right)=\left\{ \begin{array}{cc} 1 & x\geq 0\\ 0 & x<0 \end{array}\right.$$

(4) $$f_{2}\left(x\right)=\left\{ \begin{array}{cc} 1 & x>0\\ 0 & x\leq 0 \end{array}\right.$$

A motion event can then be identified using the acceleration at the *x*- and *y-*axes, where Δ*t* is the sampling period. The subscript *x* or *y* at *a*(*n*) denotes the acceleration at the two axes. The subscript ME denotes the motion event, and operator ∨ denotes the OR operation. The flag for the motion events can be computed as follows.


(5) $$flag_{\mathrm{ME}}=f_{1}\left(\left|a_{x}\left(n\right)-a_{x}\left(n-1\right)\right|-1.3\Delta T\right)\vee f_{1}\left(\left|a_{y}\left(n\right)-a_{y}\left(n-1\right)\right|-1.3\Delta T\right)$$

If *flag*_*ME*_ is one, the signals encounter a motion event; otherwise, zero. Once a first true value of the flag appears, the timing of the motion event starts and is stored/defined as *T*_*m*_. The motion event is ended when the flag remains false for over 20 s. The ending time is identified as the last sample when *flag*_*ME*_ is true.

Another flag, *flag*_*MA*_, is introduced to identify the existence of MAs, which is defined as follows.


(6) $$flag_{\text{MA}}=f_{1}\left(T_{m}-1\right)$$

*T*_*m*_ is the starting time of a motion event. The baseline of fNIRS signals, *Avg*, is defined as the average of the signal amplitudes before and after *T*_*m*_. To avoid disturbance during the motion event, we marked 5 s before *T*_*m*_ as *T*_*before*_ and 5 s after *T*_*m*_ as *T*_*after*_. The amplitudes of the signals before *T*_*m*_, *Avg*_*before*_, and those after *T*_*m*_, *Avg*_*after*_, are calculated as follows.


(7) $$Avg_{\text{before}}=\text{mean}\left({z\left(n\right)|}_{T_{\text{before}}-15\leq n<T_{\text{before}}}\right)$$

(8) $$Avg_{\text{after}}=\text{mean}\left({z\left(n\right)|}_{T_{\text{after}}<n\leq T_{\text{after}}+15}\right)$$

*flag*_*BS*_ is a flag identifying baseline shifts in motion events. Specifically, one for baseline shifts, and zero for no shift.


(9) $$flag_{\text{BS}}=f_{2}\left(\left|Avg_{\text{before}}-Avg_{\text{after}}\right|-2.6\sigma_{\text{before}}\right)$$

(10) $$\sigma_{\text{before}}=\text{std}\left({z\left(n\right)|}_{T_{\text{before}}-15\leq n<T_{\text{before}}}\right)$$

The function std(⋅) computes the standard deviation of its input.

The correction procedure only applies to baseline shift segments. Moreover, a flag for correction, *flag*_*crr*_, is introduced. If its value is one, a correction of signals will be conducted; otherwise, it is zero. Moreover, operator ∧ denotes the AND operation. The *flag*_*crr*_ can be computed as follows.


(11) $$flag_{\text{crr}}=f_{1}\left(N_{\text{ch}}\left({flag_{\text{BS}}|}_{flag_{\text{BS}}=1}\right)-2\right)\vee f_{1}\left(N_{\text{wv}}\left({flag_{\text{BS}}|}_{flag_{\text{BS}}=1}-2\right)\right)\wedge flag_{\text{ME}}$$

where *N*_*ch*_(⋅) denotes the number of channels satisfying the condition specified in the input, and *N*_*wv*_(⋅) denotes the number of wavelengths satisfying the input. When *flag*_*crr*_ is one, *z*(*n*) is corrected as follows.


(12) $${\hat{z}\left(n\right)|}_{n\mathrm{after}T_{m}}=\frac{Avg_{\text{before}}}{Avg_{\text{after}}}z\left(n\right)$$

(13) $${\hat{z}\left(n\right)|}_{n\mathrm{inside}T_{m}}=A_{\text{before}}$$

The ABAMAR solution is applied to optical intensities, optical densities, and concentration changes. It can efficiently suppress step-like artifacts. However, the signal details during motion events will be lost owing to the correction method. Moreover, empirical constants, such as 1.3 *g*/s in Eq. (5) (*g* denotes the gravitational acceleration of 9.81 m/s^2^) and 2.6 in Eq. (9), may need to be updated for tasks other than sleeping monitoring. Some researchers have proposed an AMARA, which is an improvement of ABAMAR (Metz et al., 2015), by combining the movement artifact reduction algorithm (MARA; see section “Spline interpolation”) and ABAMAR.

### Linearly polarized light-based solution

#### Multidistance optode arrangement technique

An optical model of light transmission between a source and a detector is developed using light transmittance (Yamada et al., 2015). Here, the light transmittances of the source-scalp gap, detector-scalp gap, and head tissue at time instance *n* are denoted by *r*_*s*_(*n*), *r*_*d*_(*n*), and *R*(*n*), respectively. Moreover, the light intensity emitted by the source is denoted by *I*_0_, the light intensity reflected by the hair is denoted by *I*_1_(*n*), and the light intensity scattered by the head tissue is denoted by *I*_2_(*n*). The optical density can then be computed as follows.


(14) $$\Delta A\;\left(n\right)=-\frac{I_{1}\left(n\right)+I_{2}\left(n\right)}{I_{1}\left(0\right)+I_{2}\left(0\right)}$$

$$\;=-\log\frac{I_{1}\left(n\right)+I_{0}r_{s}\left(n\right)R\left(0\right)r_{d}\left(n\right)}{I_{1}\left(0\right)+I_{0}r_{s}\left(0\right)R\left(0\right)r_{d}\left(0\right)}$$

$$\;-\log\frac{I_{1}\left(n\right)+I_{0}r_{s}\left(n\right)R\left(n\right)r_{d}\left(n\right)}{I_{1}\left(n\right)+I_{0}r_{s}\left(n\right)R\left(0\right)r_{d}\left(n\right)},$$

where


(15) $$I_{0}=I_{1}\left(n\right)+I_{2}\left(n\right)$$

The MA removal solution includes two steps: Step 1 involves suppressing *I*_1_(*n*), and Step 2 involves attenuating the first term in the model.

Polarized optical films are attached to the source and detector in an orthogonal direction to suppress *I*_1_(*n*). Light reflection does not change the polarization of light, but scattering will change; therefore, only scattered light can be captured by the detector, that is, *I*_1_(*n*) = 0. Thus, Eq. (14) can be reduced to the following form:


(16) $$\Delta A\;\left(n\right)=-\log\frac{r_{s}\left(n\right)r_{d}\left(n\right)}{r_{s}\left(0\right)r_{d}\left(0\right)}-\log\frac{R\left(n\right)}{R\left(0\right)}$$

**FIGURE 3:**
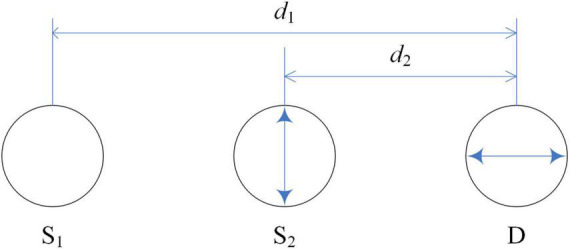
Optode arrangement for multidistance optode arrangement technique.

Step 2 is to cancel out the first term because it is independent of the hemodynamic changes. The optode arrangement is depicted in Figure 3. Accordingly, the method assumes that if two unidirectional inline channels have a small distance difference (two sources and one detector), their concentration changes will have similar temporal patterns. The hemodynamic changes contribute to the second term in Eq. (16), so according to the modified Beer–Lambert law, we obtain the following equation:


(17) $$-\log\frac{R\left(n\right)}{R\left(0\right)}=DPF\cdot d\Delta \mu$$

where *DPF* denotes the differential pathlength factor, *d* indicates the source-detector distance, and Δ*μ* corresponds to the absorption coefficient change in the gray matter. Moreover, the light transmittances of the source-scalp gaps for both channels and the detector-scalp gap for the detector are denoted by *r*_*s*1_(*n*), *r*_*s*2_(*n*), and *r*_*d*_(*n*), respectively. *R*_1_(*n*) and *R*_2_(*n*) denote the transmittances of the head tissues for the two channels. We can obtain the following equation from Eq. (17) by weighted subtraction of the optical densities of the two channels.


(18) $$\Delta A_{1}\left(n\right)-k\Delta A_{2}\left(n\right)=-\log\frac{r_{s1}\left(n\right)r_{d}\left(n\right)}{{\left(r_{s2}\left(n\right)r_{d}\left(n\right)\right)}^{k}}$$

$$\;-\log r_{s1}\left(0\right)r_{d}\left(0\right)+k\log r_{s2}\left(0\right)r_{d}\left(0\right)$$

$$\;-\log\frac{R_{1}\left(n\right)}{R_{1}\left(0\right)}+k\log\frac{R_{2}\left(n\right)}{R_{2}\left(0\right)}$$

$$\;=-\log\frac{r_{s1}\left(n\right)r_{d}\left(n\right)}{{\left(r_{s2}\left(n\right)r_{d}\left(n\right)\right)}^{k}}$$

$$\;+C+\left(DPF_{1}d_{1}-kDPF_{2}d_{2}\right)\Delta \mu \left(n\right).$$

The constant *k* (approximately one) depends on the wavelength, and *C* is a constant that depends on the initial installation of the device. When the two sources and the detector are fixed relatively well, *r*_*s*1_(*n*) and *r*_*s*2_(*n*) will be consolidated to a similar value. Thus, the first term tends to be zero.

The solution above was evaluated on a hairy phantom, and its hardware solution in the first step inspired a creative solution to the hair-blocking problem encountered while using fNIRS devices. The validity of the second step is decided by the approximation that *k* = 1, whereas in actual measurement, *k* may occasionally become negative (Yamada et al., 2015). Besides, the optical model neglects detection noise and the angular fluctuation of optodes. The multidistance optode arrangement technique is applied to optical density and is available for real-time monitoring. The solution in Step 1 (using polarized optical film) can attenuate hair-reflected light.

## Signal Processing-Based Techniques

### Wiener filter

The Wiener filter approach is the first to remove motion artifacts without incorporating additional hardware devices (Izzetoglu et al., 2005; Orihuela-Espina et al., 2010; Li L. et al., 2021). The technique assumes that the measured fNIRS signals are a simple addition between the actual fNIRS signals, *x*(*n*), and motion artifacts, *v*(*n*). Moreover, it is assumed that *x*(*n*) and *v*(*n*) are stationary and uncorrelated.


(19) corr(x(n),v(n))=0

Consequently, the Wiener filter, *g*(*n*), minimizes the mean square error between *x*(*n*) and $\hat{x}$(*n*), that is,


(20) $$\min\left(\text{E}\left[e{\left(n\right)}^{2}\right]\right)=\min\left(\text{E}\left[{\left(x\left(n\right)-\hat{x}\left(n\right)\right)}^{2}\right]\right)$$

Therefore, the optimum filter can be obtained using the orthogonality principle and simplified using Eq. (19) as follows.


(21) corr[e(n),x(n)+v(n)]=corr[x(n),x(n)]

                                                                       -g(n)*corr[x(n)+v(n),

                                                     x(n)+v(n)]

                  =0.

We obtain the Fourier transform of the Wiener filter by converting Eq. (21) into the frequency domain using the Fourier transform, which is as follows.


(22) $$G\left(w\right)=\frac{p_{x}\left(w\right)}{p_{x}\left(w\right)+p_{v}\left(w\right)}$$

where *p*_*x*_(*w*) and *p*_*v*_(*w*) denote the power spectral densities (PSDs) of the actual fNIRS signals and motion artifacts.

In application, a prior experiment is required to determine the PSDs of *g*(*n*) by calculating the values of *x*(*n*) and *v*(*n*). Subsequently, we can determine *g*(*n*) on a time scale using the inverse Fourier transform and apply it to new experimental data.

The Wiener filter is the first attempt to remove motion artifacts without a reference signal from additional hardware devices, such as accelerometers. With the *g*(*n*) determined, the filter can be implemented for online applications. However, it requires prior knowledge of the PSDs of *x*(*n*) and *v*(*n*), which makes initial calibration more complex (a particularly designed paradigm is needed). The idea of building the filter model from a prior experiment inspired later research on motion artifact removal techniques.

### Kalman filter

The Kalman filter approach was also proposed based on the general idea of the Wiener filter but as a different model (Izzetoglu et al., 2010). The motion artifact-free fNIRS signal, *x*(*n*), was modeled using an autoregressive (AR) model. An AR model of order *p* can be written as


(23) $$x\left(n\right)=\sum_{i=1}^{p}a_{i}x\left(n-i\right)$$

With the motion artifact-free data from the prior experiment, the parameters of the AR model, *a*_*i*_, *i* = 1, …, *p*, can be determined using the Yule-Walker equations. Therefore, the process equation for the Kalman filter has the following form.


(24) $$\text{ϕ}\left(n\right)=A\text{ϕ}\left(n-1\right)+{\text{ω}}_{n},\text{ϕ}\left(n\right)={\left[\begin{array}{c} x\left(n\right)\;\text{⋯}\;x\left(n-p+1\right) \end{array}\right]}^{T}$$

where ϕ(*n*) is composed of *p* motion artifact-free fNIRS signals, and ω*_*n*_* denotes the zero-mean noises in the AR model with an error covariance matrix *Q*. Matrix *A* can be obtained using Eq. (23) as follows.


(25) $$A=\left[\begin{array}{cccc} a_{1} & \text{⋯} & a_{p-1} & a_{p}\\ 1 & \text{⋯} & 0 & 0\\ \vdots & \text{⋯} & \text{⋯} & \vdots \\ 0 & \text{⋯} & 1 & 0 \end{array}\right]$$

The measurement equation can be written as follows.


(26) $$z\left(n\right)=C\text{ϕ}\left(n\right)+\nu_{n}$$

(27) $$C=\underset{p\mathrm{elements}}{\underbrace{\left[\begin{array}{cccc} 1 & 0 & \text{⋯} & 0 \end{array}\right]}}$$

where ν*_*n*_* denotes the measurement noise (such as the motion artifact) with an error covariance matrix *R*. *z*(*n*) denotes the motion artifact corrupted signal.

Subsequently, Eqs. (24) and (26) form the state-space model for the Kalman filter, see Table 4. The minus sign in the superscript denotes the prior estimate of a variable. Subsequently, the motion-free fNIRS signal can be obtained using the Kalman filter (Wan and Nelson, 2001). The Kalman filter method can be applied to any online application of optical intensities, optical densities, and concentration changes, and in the Kalman filter theory, both ω*_*n*_* and ν*_*n*_* are assumed to be zero-mean Gaussian white noise (Huang and Hong, 2021; Haghighi and Pishkenari, 2021; Li B. A. et al., 2021; Sun and Zhao, 2021; Yang et al., 2021; Lv et al., 2021; Tang et al., 2020; Pham et al., 2021). However, it is not the case for ν*_*n*_* (motion artifacts do not observe the zero-mean Gaussian distribution). It may degrade the filter’s performance (Zhou et al., 2017). Moreover, matrices *A* and *C* were fixed once determined by the Yule-Walker method. Therefore, further development of the algorithm focuses on; (i) the compensation of the instrumental noises (Amian and Setarehdan, 2013) and (ii) adaptive adjustment of *A* and *C*, or using a more sophisticated and nonlinear model in place of Eqs. (24) and (26) (Dong and Jeong, 2018).

**TABLE 4:** Kalman filter algorithm.

**Initialization:**
$\hat{\text{ϕ}}\left(0\right)=E\left(\text{ϕ}\left(0\right)\right)$,
*P*(0) = E((ϕ(0) − E(ϕ(0)))(ϕ(0) − E(ϕ(0)))^*T*^).
Computation: For *n* = 1, 2, …, compute:
**State estimate propagation**
${\hat{\text{ϕ}}}^{-}\left(n\right)=A\hat{\text{ϕ}}\left(n-1\right)$.
**Error covariance propagation**
*P*^−^ (*n*) = *AP*(*n* − 1) *A^T^* + *Q*.
**Kalman gain matrix**
*G*(*n*) = *P*^−^ (*n*)*C^T^* (*CP*^−^ (*n*)*C^T^* + *R*)^−1^ .
**State estimate update**
$\hat{\text{ϕ}}\left(n\right)={\hat{\text{ϕ}}}^{-}\left(n\right)+G\left(n\right)\left(z\left(n\right)-C{\hat{\text{ϕ}}}^{-}\left(n\right)\right)$.
**Error covariance update**
*P* (*n*) = (*Id* − *G*(*n*)*C*)*P*^−^ (*n*).

Additionally, the state-space model for the Kalman filter method is not unique. A typical example is the incorporation of the autoregressive iterative robust least-squares model (AR-IRLS), the general linear model (GLM), and two linear Kalman filters (Barker et al., 2016). A GLM and an AR-IRLS replaced the state-space model in the Kalman filters. The GLM was introduced to describe the dynamics of hemodynamic responses and physiological noises. The AR-IRLS was introduced as compensation for the MAs in the signals. A difference between the GLM-based method and the AR model-based method is that the GLM-based method requires the information of experimental paradigms, while the AR model-based method does not. Despite the significant adaptation ability of the Kalman filter regarding its state-space model, its applications are limited due to the effort required to set its initial parameters (e.g., the error covariance matrices for the state and the observation).

### Spline interpolation

**FIGURE 4:**
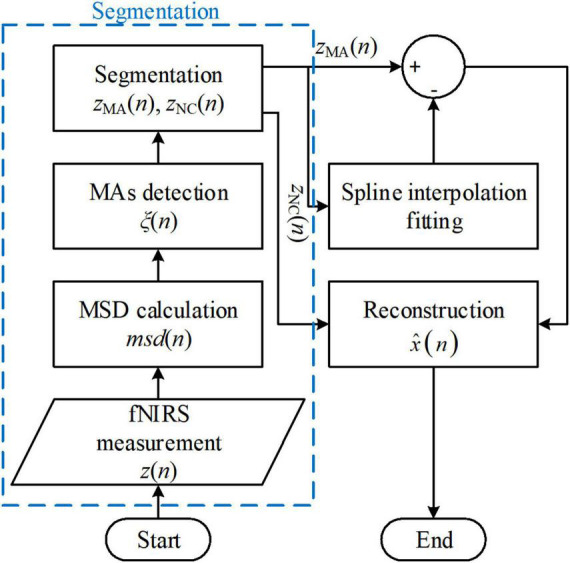
Flowchart of the movement artifact reduction algorithm (MARA) algorithm. The process blocks in the blue box are one of the reasons that limit the solution’s online application.

The spline interpolation method was first proposed by Scholkmann’s group and is referred to as the MARA (Scholkmann et al., 2010; Selb et al., 2015; Lee et al., 2021). The method made two fundamental assumptions: (i) The measured fNIRS signal is a linear addition of the motion artifacts and the motion-free fNIRS signal, and (ii) in the motion-corrupted segments in the signal, the motion artifact component dominates the measured fNIRS signal. Therefore, the proposed MARA comprised two parts: (i) Motion artifact detection and segmentation, and (ii) motion artifact removal. A flowchart of MARA is illustrated in Figure 4. The spline interpolation method encompasses six processing steps. First, the moving standard deviation (MSD) is calculated within a moving time window of *W* samples and stored as *msd*(*n*).


(28) $$msd\left(n\right)=\frac{1}{W}\sqrt{\sum_{i=-k}^{k}n+i-\frac{1}{W}{\left(\sum_{i=-k}^{k}z\left(n+i\right)\right)}^{2}},W=2k+1,k\in {\mathbb{N}}^{*}$$

where N* is the set of natural numbers.

We can determine the start and end points of the motion artifacts and store the indices of the corresponding samples in a vector *ξ*(*n*) by comparing with the MSD [i.e., *msd*(*n*) in Eq. (28)] with a user-defined threshold value *T*. If the MSD is smaller than *T*, the corresponding *msd*(*n*) will be assigned as zero. The start points and endpoints of motion artifacts can be extracted by considering the first and last samples of non-zero values in *msd*(*n*). Next, let us suppose that there are *L* segments of motion artifacts and let the motion artifact segments, *z*_*MA*_(*n*), and the non-corrupted segments, *z*_*NC*_(*n*), be expressed, respectively, as


(29) $$z_{\text{MA}}\left(n\right)=\left\{z_{\text{MA},1}\left(n\right),\text{⋯},z_{\text{MA},L}\left(n\right)\right\}$$

(30) $$z_{\text{NC}}\left(n\right)=\left\{z_{\text{NC},1}\left(n\right),\text{⋯},z_{\text{NC},L}\left(n\right)\right\}$$

Using Eqs. (29) and (30), the measured fNIRS signals can be segmented into non-corrupted segments and motion artifact segments.

In the next part, the spline interpolation method corrects motion artifact segments. Because the motion artifact components dominate the MA segments, the spline interpolation fitting of *z*_*MA*_(*n*) can be viewed as the motion artifact component. The difference between *z*_*MA*_(*n*) and its spline interpolation fitting is stored as *z*_*diff*_(*n*):


(31) $$z_{\text{diff}}\left(n\right)=\left\{z_{\text{diff},1}\left(n\right),\text{⋯},z_{\text{diff},L}\left(n\right)\right\}$$

Because *z*_*NC*_(*n*) and *z*_*diff*_(*n*) may have different magnitude levels, the final step involves correcting the signal levels for the entire time series. Each segment is parallel-shifted according to the mean of the previous segment and that of the target segment. Two empirical constant thresholds, α = 3^–1^ Hz^–1^⋅*f*s and *β* = 2 Hz^–1^⋅*f*s (where the variable *f*s denotes the sampling frequency), were chosen for comparison. The detailed shifting rules are listed in Table 1 in Scholkmann et al. (2010).

The spline-interpolation method is used widely for offline analysis in fNIRS studies. It has also been included in some open-source toolboxes, such as HomER2 and NIRSLAB (Balardin et al., 2017). Moreover, the method applies not only to the optical intensities and optical densities but also to concentration changes. However, both the segmentation procedure (the procedures in the blue box in Figure 4) and the parallel-shifting procedure (in the reconstruction process) increase the difficulty for online filtering applications. Moreover, the filter performance depends on artifact detection results (Brigadoi et al., 2014). Some variations of the spline interpolation method have also been proposed (Jahani et al., 2018; Zhou et al., 2021).

### Wavelet-based method

The wavelet-based method eradicates motion artifacts by removing the corresponding wavelet coefficients (Molavi and Dumont, 2012; Pinti et al., 2015), without requiring auxiliary devices. The method made the same assumption as the Wiener filter case, that is, *z*(*n*) = *x*(*n*) + *v*(*n*). Based on discrete wavelet transform (DWT), fNIRS signals can be expanded as follows.


(32) $$z\left(n\right)=\sum_{k}a_{i_{0}k}\varphi_{i_{0}k}\left(n\right)+\sum_{i=i_{0}}^{\infty }\sum_{k}b_{ik}\psi \left(n\right)$$

where *i* denotes the dilation parameter, *k* indicates the translation parameter, and *i*_0_ denotes the coarsest scale. The scaling function φ_*i*_0_*k*_ and the wavelet function ψ*_*ik*_* are as follows.


(33) $$\varphi_{i_{0}k}\left(n\right)=2^{i_{0}/2}\Phi \left(2^{i_{0}}n\text{-}k\right)$$

(34) $$\psi_{ik}\left(n\right)=2^{i/2}\Psi \left(2^{i}n\text{-}k\right)$$

The functions Φ(*n*) and Ψ(*n*) correspond to the mother scaling function and mother wavelet function, respectively. With the fast wavelet transform, the coefficients of the DWT of *z*(*n*) become


(35) $$a_{i_{0}k}=\sum_{l=0}^{N-1}h\left(l-2k\right)z\left(l\right),k=0,\ldots \;,2^{i}-1$$

(36) $$b_{ik}=\sum_{l=0}^{N-1}g\left(l-2k\right)z\left(l\right),i=i_{0},\ldots \;,J-1$$

where *N* denotes the number of samples in the signal and *N* = 2*^J^*. *g*(*n*) and *h*(*n*) denote the wavelet filter bank high- and low-pass filters, respectively. Therefore, Eqs. (35) and (36) can be written in the matrix form as follows.


(37) $$W=\text{Ω}Z,Z={\left[z\left(0\right)\;\text{…}\;z\left(N-1\right)\right]}^{T}$$

where *W* = [*B*_1_, *B*_2_, …, *B*_*J*–1_, *A*_*i*0_]*^T^, B*_*i*_ = [*b*_*i*0_, …, *b*_*i*(2∧_*_*i*_*_–1)_], *A*_*i*0_ = [*a*_*i*00_, …, *a*_*i*0(2∧_*_*i*_*_0–1)_]. Ω is an *N* by *N* DWT matrix. For any element *w*_*ik*_ in the wavelet coefficient vector *W*, the probability of observing its value can be written as


(38) $$p_{ik}=2\left(1-NCDF\left(\frac{\left|w_{ik}\right|}{\hat{\sigma }}\right)\right)$$

where *NCDF*(⋅) is a normal cumulative distribution function. The estimated standard deviation of *w*_*ik*_ can be approximated empirically as


(39) $${\hat{\sigma }}_{i}=\frac{\text{Median}\left(\left|W_{i}\right|\right)}{0.6745}$$

where *W*_*i*_ denotes *B*_*i*_ or *A*_*i0*_ according to the value of *i*. Given a probability threshold α, if *p*_*ik*_ < α, we consider that the corresponding wavelet coefficient *w*_*ik*_ is dominated by artifacts and set to zero. The filtered signals can be obtained from the updated wavelet coefficient vector as


(40) $${\left[\hat{x}\left(0\right)\;\text{…}\;\hat{x}\left(N-1\right)\right]}^{T}={\text{Ω}}^{T}\hat{W}$$

The hat over *x*(⋅) denotes the filtered fNIRS signal, and the hat over *W* denotes the updated wavelet coefficient vector after thresholding.

Similar to the spline interpolation method, the wavelet-based method can be applied to optical intensities, optical densities, and concentration changes. Initially, the wavelet-based method was first introduced as an offline artifact removal algorithm. It may be adjusted to be an online method using the windowing technique; however, its filtering performance will be degraded. Several variations of this method have also appeared in recent years (Chiarelli et al., 2015; Shukla et al., 2018; Perpetuini et al., 2021). Gao Y. et al. (2022) addressed that the tuning of the probability threshold is crucial, and Wei et al. (2018) designed a dual-threshold structure to improve the performance of the wavelet-based method.

### Correlation-based signal improvement method

Based on the negative correlation between measured oxyhemoglobin (HbO) and deoxyhemoglobin (HbR) concentrations, the correlation-based signal improvement (CBSI) method was proposed in 2010 (Cui et al., 2010; Haeussinger et al., 2014). The algorithm made three fundamental assumptions: (i) HbO concentrations are strictly negatively correlated with HbR concentrations (close to –1); (ii) motion artifacts have identical effects on HbO and HbR, subject to a constant factor; and (iii) motion artifacts are not correlated to the actual concentration changes. The measured concentration changes were modeled as follows.


(41) $$\begin{array}{c} z_{\text{HbO}}\left(n\right)=x_{\text{HbO}}\left(n\right)+\chi v,\\ z_{\text{HbR}}\left(n\right)=x_{\text{HbR}}\left(n\right)+v, \end{array}$$

where *v* denotes the motion artifact of the HbR signals, and χ is a constant. Based on the first assumption, we can reasonably obtain


(42) $$x_{\text{HbO}}\left(n\right)=-\beta x_{\text{HbR}}\left(n\right)$$

*β* denotes the free ratio accounting for the magnitude difference between HbO and HbR. Equations (41) and (42) can be used to obtain the following:


(43) $$\begin{array}{c} v\left(n\right)=\frac{z_{\text{HbO}}\left(n\right)+\beta z_{\text{HbR}}\left(n\right)}{\chi +\beta },\\ x_{\text{HbO}}\left(n\right)=\frac{\beta }{\chi +\beta }\left(z_{\text{HbO}}-\chi z_{\text{HbR}}\right). \end{array}$$

Based on the third assumption, we set the correlation between *v* and *x*_*HbO*_ to zero, yielding


(44) $$\sum_{n}z_{\text{HbO}}{\left(n\right)}^{2}+\left(\beta -\chi \right)\sum_{n}z_{\text{HbO}}\left(n\right)z_{\text{HbR}}\left(n\right)-\chi \beta \sum_{n}z_{\text{HbR}}{\left(n\right)}^{2}=0$$

We assumed that χ = *β*. Then, Eq. (44) can be reduced to


(45) $$\chi =\frac{\sigma_{\text{HbO}}}{\sigma_{\text{HbR}}}$$

where σ_*HbO*_ and σ_*HbR*_ denote the standard deviations of *z*_*HbO*_ and *z*_*HbR*_, respectively. Therefore, the final CBSI filter is


(46) $$\begin{array}{c} x_{\text{HbO}}\left(n\right)=\frac{1}{2}\left(z_{\text{HbO}}\left(n\right)-\chi z_{\text{HbR}}\left(n\right)\right),\\ x_{\text{HbR}}\left(n\right)=-\frac{1}{\text{α}}x_{\text{HbO}}\left(n\right). \end{array}$$

Once χ is decided based on prior-experimental data, the CBSI filter can be easily implemented for online applications using Eq. (46). As mentioned in (Cui et al., 2010), CBSI can be applied after an exponential moving average (EMA) filter to facilitate real-time analysis. The behavior of an EMA filter is similar to an online version of a band-pass filter. The EMA is described as follows.


(47) $$\begin{array}{c} L\left(n\right)=\frac{1}{W_{\text{L}}}z\left(n\right)+\left(1-\frac{1}{W_{L}}\right)L\left(n-1\right),\\ S\left(n\right)=\frac{1}{W_{\text{S}}}\sum_{k=0}^{W_{s}}z\left(n-k\right),\\ x\left(n\right)=S\left(n\right)-L\left(n\right). \end{array}$$

*L*(*n*) denotes the signal filtered by a large-window moving average (window size as *W*_*L*_), and *S*(*n*) denotes the signal filtered by a moving average of window size *W*_*S*_.

Owing to the fundamental assumptions of the filter, the CBSI filter can only be applied to concentration changes. However, the negative correlation assumption between the HbO and HbR concentrations may not always be true for experimental data. Typically, the CBSI method is used for comparison in the literature (Janani and Sasikala, 2017; Reyes et al., 2018; Sherafati et al., 2020).

### Principal component analysis

The solution based on principal component analysis (PCA) was developed from a multivariate method to remove physiological noises. The PCA-based method, entitled targeted PCA (tPCA), is similar to spline interpolation, except for using PCA instead of spline interpolation to fit the noisy segments in the signals (Yucel et al., 2014a). In this subsection, we subsequently explain the mechanism of Zhang’s method and tPCA.

In accordance with the regular PCA method, the systemic spatial interference subspace (the actual hemodynamic responses) dominates in the baseline over the measured concentration changes and it spans during the stimulation periods for the first few components. The measured fNIRS signals are first converted to HbO and HbR concentration changes and are band-pass filtered. The filtered data from a chosen channel set are formatted into two *N*_*ch*_ × *N* matrices, *Z*_*HbO*_ and *Z*_*HbR*_, where *N*_*ch*_ denotes the number of channels and *N* is the number of time samples. When segmented into two parts according to the baseline and stimulation periods, the two matrices turn out to be as follows.


(48) $$Z_{\text{HbO}}=\left[\begin{array}{cc} Z_{\text{HbO},\text{base}} & Z_{\text{HbO},\text{stim}} \end{array}\right]$$

(49) $$Z_{\text{HbR}}=\left[\begin{array}{cc} Z_{\text{HbR},\text{base}} & Z_{\text{HbR},\text{stim}} \end{array}\right]$$

For simplification, we omit the subscripts HbO and HbR, primarily because the filtering operations are identical for both chromophores. Accordingly, the spatial correlation matrices for the baseline signals are as follows.


(50) $$C_{\text{base}}=1/N\cdot Z_{\text{base}}Z_{\text{base}}^{T}$$

The eigen-values and the eigenvectors can be obtained by the eigen-decomposition of spatial correlation matrices:


(51) $$C_{\text{base}}=U_{\text{base}}\Sigma_{\text{base}}U_{\text{base}}^{T}$$

where the rows of *U*_*base*_ contains the eigenvectors, and Σ_*base*_ contains the eigenvalues in its diagonal. Based on the dominant interference subspace assumption, we assume that the first *r* spatial eigenvectors span in the stimulation period as *U*_*base*,_*_*r*_*, an *N*_*ch*_ by *r* matrix padding by zeros. Accordingly, the actual hemodynamic responses will be the projection of the measured fNIRS signals onto the orthogonal subspace of the first *r* eigenvectors. The superscript ^⊥^ denotes the orthogonal subspace. Then, the filtered fNIRS signals were


(52) $$X_{\text{stim}}=\left(Id-U_{\mathrm{base},r}U_{\mathrm{base},r}^{T}\right)Z_{\text{stim}}=U_{\mathrm{base},r}^{⊥}U_{\mathrm{base},r}^{⊥T}Z_{\text{stim}}$$

where *X*_*stim*_ denotes the filtered fNIRS signal and matrix *Id* is an *N*_*ch*_ by *N*_*ch*_ identity matrix. Finally, we can block the average *X*_*stim*_ around the stimulus to improve the signal-to-noise ratio (SNR).

To remove MAs, tPCA applies the multivariate PCA method on MA segments. The eigenvectors in *U*_*base*_ are ranked in decreasing order of the percent variance of eigenvalues in Σ_*base*_. The number of spatial eigenvectors span, *r*, is the minimum integer obtaining not less than 97% percent variance. Then, the filtered fNIRS signals of the segments are stitched back into the original signals after shifting the average of adjacent data segments (identical to MARA). The processing procedures are repeated twice or three times until there is no further improvement in the results.

Targeted PCA applies to optical densities. Due to the iteration in MA identification, the method cannot apply to online filtering. tPCA can remove both spike-like MAs and baseline shifts. It can better suppress high-frequency MAs than MARA (Yucel et al., 2014a). However, during application, the performance of tPCA may degrade if the MAs are not present in multiple channels (Brigadoi et al., 2014). The method requires multiple channels, which is not an appropriate choice when the number of channels is limited.

### Multi-channel regression

The idea of the method is to subtract a best-fit linear combination of the noise references from each signal (Robertson et al., 2010). For a *k*-channel regression, the measured signal is modeled as follows.


(53) z=aH+x

where *z ϵ* ℝ^1 ×^ *^N^, a ϵ* ℝ1 × *k, H ϵ* ℝ^(^*^k^*^ + 1) ×^*^N^, x ϵ* ℝ^1 × *N*^. The row vector, *a*, is the weight for each reference channel in *H*. The regressor *H* is defined as follows.


(54) $$H=\left[\begin{array}{ccc} 1 & \text{⋯} & 1\\ z_{r1}\left(1\right) & \text{⋯} & z_{r1}\left(N\right)\\ \vdots & \vdots & \vdots \\ z_{rk}\left(1\right) & \text{⋯} & z_{rk}\left(N\right) \end{array}\right]$$

where *z*_*ri*_ (*i* = 1, 2, …, *k*) is the measurement of the *i*th reference channel. It is recommended to take co-located channels as reference channels in priority. Therefore, *a* can be estimated using the least-squares method.


(55) $$a={\left(H^{T}H\right)}^{-1}H^{T}z$$

The motionless signal, *x*, can be obtained by subtracting *aH* from *z*.

The multi-channel regression method does not require an auxiliary device and applies to optical intensities, optical densities, and concentration changes. It was first designed as an offline filtering method, but it can apply to online applications simply by replacing the least-square estimation procedure with recursive least-square estimation. When different types of artifacts affect collocated channels differently, the multi-channel regression method may not remove MAs entirely.

### Independent component analysis

Independent component analysis (ICA) can decompose a signal to the weighted sum of multiple independent sources statistically (Kohno et al., 2007; Santosa et al., 2013). Therefore, the ICA solutions belong to blind source separation methods (von Luhmann et al., 2019). The key to removing MAs using ICA is how to select the components relevant to motionless signals or MAs. Kohno et al. (2007) suggested identifying the components related to artifacts using the coefficient of spatial uniformity (CSU).


(56) $$CSU_{j}=\left|\frac{{\bar{s}}_{j}}{\text{std}\left(s_{j}\right)}\right|$$

Additionally, Santosa et al. (2013) estimated motionless signals by weighted reconstructing from independent components according to the *t*-values between each component and the desired hemodynamic response. The desired hemodynamic response is the convolution between the canonical hemodynamic response and the experimental paradigm (a boxcar function).

The ICA applies to optical intensities, optical densities, and concentration changes. The input requirements may vary according to the reconstruction strategy. Some researchers concluded that the performance of ICA was not satisfactory in some applications because fNIRS signals contained both non-instantaneous and non-constant coupling, correlated noise, and source dependencies (von Luhmann et al., 2019).

### Temporal derivative distribution repair

The temporal derivative distribution repair (TDDR) is an online filtering method based on the temporal derivative of fNIRS signals. Three basic assumptions were made for this method: Non-motion fluctuations are assumed to be normally distributed; most fluctuations are independent of motion artifacts; and the derivatives of motion artifacts dominate in the derivatives of fNIRS signals when they are present (Fishburn et al., 2019). Therefore, the algorithm can be divided into five steps.

Step 1: For time instance *n*, the variation in the fNIRS signal can be calculated as follows.


(57) Dz(n)=z(n)-z(n-1)

where the *D* before *z*(*n*) denotes the temporal derivative of the measured signal at instance *n*.

Step 2: The observation weight *w*(*n*), corresponding to *Dz*(*n*), is initialized as one.

Step 3: The observation weight is adaptively updated using a robust estimator, M-estimator, and Tukey’s biweight function:


(58) $$w\left(n\right)=\left\{ \begin{array}{cc} {\left(1-\sigma_{s}{\left(n\right)}^{2}\right)}^{2} & \sigma_{s}\left(n\right)<1,\\ 0 & \mathrm{otherwise}, \end{array}\right.$$

where σ_*s*_(*n*) denotes the scaled deviation at instance *n*. An estimate of σ_*s*_(*n*) can be obtained from the weighted mean of the fluctuations, the absolute residuals of the temporal derivative, and the median absolute residual scale for the normal distribution. Let μ denote the weight mean of fluctuations, *res*(*n*) denote the absolute residuals of temporal derivatives, and σ(*n*) represent the median absolute residuals scale. Subsequently, we can obtain the following:


(59) $$\mu =\frac{1}{\sum_{i=1}^{n}w\left(i\right)}\sum_{i=1}^{n}w\left(i\right)Dz\left(i\right)$$

(60) res(n)=|Dz(n)-μ|

(61) σ=1.4826⋅median(res)

Repeat (54)–(57) and consider σ_*s*_(*n*) = σ(*n*) until μ converges.

Step 4: The filtered temporal derivative can be obtained using μ and *w*(*n*) obtained in Step 3.


(62) Dx(n)=w(n)(Dz(n)-μ)

Step 5: The final filtered signal is the integration of corrected temporal derivatives.


(63) $$x\left(n\right)=\sum_{i=1}^{n}Dx\left(n\right)$$

Importantly, the filter can be implemented offline and online. The only difference is that when implemented online, the sum of *w*(*n*) and *w*(*n*)*Dz*(*n*) should be stored in each loop to facilitate the calculation in Eq. (59). The TDDR algorithm can be used for concentration changes, optical intensities, and optical densities. The performance and computation time of the TDDR depend on the convergence criterion in Step 3. In particular, in Eq. (61), the iterative application of the median operator may result in a significant computation time. Furthermore, as the total number of data increases, the computation time increases according to regarding the complexity of the sorting algorithm in the median filter. Fishburn’s group claimed that 130 ms were required to process a dataset of 20 Hz (50 ms in period) (Fishburn et al., 2019). A long computation time may lead to a loss in data transfer or a delay in the data output. In addition, high-frequency noise may degrade the performance of TDDR.

### Transient artifact reduction algorithm

The transient artifact reduction algorithm (TARA) is an offline artifact removal method. The algorithm attempts to estimate motion artifacts instead of filtering the original signal directly (Selesnick et al., 2014). Generally, motion artifacts can be divided into two types. The Type 1 artifacts are modeled as spikes, whereas Type 2 artifacts are modeled as additive step shapes. The corrupted fNIRS signals were modeled as a low-pass filter/compound noise denoising problem (LPF/CSD).


(64) $$z=x+\nu_{1}+\nu_{2}+\text{ω}$$

where ν_1_ and ν_2_ denote the two types of MAs, and ω denotes the Gaussian white noise. Besides, the desired uncorrupted fNIRS signal *x* is assumed to approximate an all-zero signal when filtered by an appropriately chosen high-pass filter, denoted by *HF*. Accordingly, when designed as a zero-phase recursive discrete-time filter, *H* can be factorized into matrices *A* and *B* such that


(65) $$HF=BA^{-1}$$

The first-order difference operator matrix *D* is defined as follows.


(66) $$D=\underset{N\mathrm{elements}}{\underbrace{\left[\begin{array}{ccccc} -1 & 1 & & & \\ & -1 & 1 & & \\ & & \ddots & \ddots & \\ & & & -1 & 1 \end{array}\right]}}$$

Inversely, the discrete-time integrator *S* is defined as the matrix satisfying *DS* = *Id*_*N* ×_ *_*N*_*, where *Id*_*N* ×_ *_*N*_* indicates an *N* by *N* identity matrix. Then, matrix *B* can be further factorized as


(67) $$B=B_{1}D$$

Subsequently, we can change the variables using the following equations.


(68) $$\nu_{1}=Au_{1}$$

(69) $$\nu_{2}=SAu_{2}$$

Type 2 MAs are expressed in Eq. (69) because their first-order difference resembles Type 1 artifacts.

The two types of artifacts can be estimated using the following procedures when the factorization matrices *A* and *B* for the desired high-pass filter, two weight parameters λ_1_ and λ_2_, and two penalty functions *r*_0_, *r*_1,_ and *r*_2_ are available.

Step 1: Input fNIRS signals *z*, three regularization parameters λ_0_, λ_1_, and λ_2_, penalty functions *r*_1_ and *r*_2_, and factorization matrices *A* and *B* for the desired high-pass filter (they can be calculated from a given degree, cutoff frequency, and length of *z*).

Step 2: The first additive terms for the estimator for both types of artifacts are as follows.


(70) $$z_{1}=B^{T}BA^{-1}z$$

(71) $$z_{2}=B_{1}^{T}BA^{-1}z$$

The vectors *u*_1_ and *u*_2_ will be initialized as zero vectors.

Step 3: Repeat the following calculation until *u*_1_ and *u*_2_ converge.


(72) $${\left[\Lambda_{0}\right]}_{n,n}={\text{λ}}_{0}/\psi_{0}\left({\left[Au_{1}\right]}_{n}\right)$$

(73) $${\left[\Lambda_{1}\right]}_{n,n}={\text{λ}}_{1}/\psi_{1}\left({\left[DAu_{1}\right]}_{n}\right)$$

(74) $${\left[\Lambda_{2}\right]}_{n,n}={\text{λ}}_{2}/\psi_{2}\left({\left[Au_{2}\right]}_{n}\right)$$

(75) $$Q_{1}=2B^{T}B+A^{T}\left(\Lambda_{0}+D^{T}\Lambda_{1}D\right)A$$

(76) $$Q_{2}=2B_{1}^{T}B_{1}+A^{T}\Lambda_{2}A$$

(77) $$g=Bu_{1}-B_{1}u_{2}$$

(78) $$u_{1}=Q_{1}^{-1}\left(z_{1}+B^{T}g\right)$$

(79) $$u_{2}=Q_{2}^{-1}\left(z_{2}+B_{1}^{T}g\right)$$

where Λ*_*i*_*, (*i* = 0, 1, or 2) is diagonal, ψ*_*i*_* = *u*_*i*_/*r*_*i*_’, [⋅]*_i,j_* denotes the element located at the *i*th row and *j*th column of the matrix, and [⋅]*_*i*_* denotes the *i*th element of the vector. The superscript prime notation, ′, is the derivative operator.

Step 4: The estimated artifacts can be obtained using Eqs. (68) and (69). Accordingly, the filtered fNIRS signals becomes the following.


(80) $$x=z-\nu_{1}-\nu_{2}$$

The TARA method is applied to optical densities, optical intensities, and concentration changes. TARA exhibits a similar performance to the wavelet-based method but is better at removing Type 2 motion artifacts. TARA is limited to Type 1 and Type 2 artifacts but is not designed for oscillatory transients.

### Dual-stage median filter

The dual-stage median filter (DSMF) method resembles an online method for motion artifact removal (Huang et al., 2022). Similar to the TARA, the design of a DSMF is based on Type 1 and Type 2 artifact categories.

The measured fNIRS signal, *z*, is first filtered using the first median filter with a window size of *W*_1_.


(81) $$x_{1}={\text{median}}_{W_{1}}\left(z\right)$$

Subsequently, the difference between *z* and *x*_1_ is filtered using the second median filter with a window size of *W*_2_.


(82) $$x_{2}={\text{median}}_{W_{2}}\left(z-x_{1}\right)$$

The output of the second median filter conserves the variations in the uncorrupted signals; however, it is biased. Therefore, the final step involves compensation of the bias using the initial time point.


(83) $$x=x_{2}+z\left(0\right)$$

*W*_1_ can be set as 8 s, while *W*_2_ as 18 s.

The DSMF is applied to optical intensities, optical densities, and concentration changes. Unlike the TDDR method, although two median filters are used in the algorithm, they are called once within fixed window sizes for each estimate. Moreover, registers can store the sorted data sequence during real-time applications, thereby reducing computation time. Therefore, a long computational time problem can be solved relatively quickly. Similar to the TARA, the DSMF is not designed for oscillatory transients.

### Convolution neural networks

The convolution neural network (CNN) was adopted for MA removal in 2022 (Gao Y. et al., 2022; Kim et al., 2022). The denoising auto-encoder (DAE) model adopted a serial structure incorporating max-pooling and up-sampling layers (Gao Y. et al., 2022). The model enabled an offline MA removal by minimizing the linear combination of mean square error (*L*_*mse*_), variance (*L*_*var*_), standard deviation (*L*_*std*_), and amplitude loss (*L*_*amp*_).


(84) $$Loss=L_{\text{mse}}+\theta_{1}\times L_{\text{var}}+\theta_{2}\times L_{\text{std}}+\theta_{3}\times L_{\text{amp}}$$

where *θ*_1_, *θ*_2_, and *θ*_3_ were set as 1, 1, and 10. The model parameters were estimated using simulated data generated from experimental data in the training process. The CNN structure is shown in Figure 1 of the reference (Gao Y. et al., 2022).

Kim et al. (2022) adopted the CNN in the U-net structure for offline MA removal. Different from the DAE model, the U-net structure accepted an input containing two feature channels.


(85) $$u\left(k\right)=\left[z\left(k\right)\;dHR\left(k\right)\right]\in {\mathbb{R}}^{N\times 2},k\in \left\{1,2,\text{…}\;,J\right\}$$

where *u*(*k*) is the input of U-net of the *k*th package, *J* is the total number of packages, and *dHR*(*k*) is the desired hemodynamic response of the *k*th package. *dHR*(*k*) is the convolution between a canonical hemodynamic response function and a stimulus function.


(86) dHR(k)=cHR*stim(k)

The U-net solution adopted the mean square errors between the ground-truth motionless fNIRS signals and the estimated signals as its loss function in the training process. The configurations of the U-net structure are shown in Figure 1 of the reference (Kim et al., 2022).

The DAE model can be applied to optical intensities for offline filtering. It takes measured data in a full-time sequence, outputs the estimated motionless signals, and is trained using simulated data from an AR model. Therefore, the quality of the simulated data has a significant impact on the filtering performance of the model.

In contrast, the U-net structured CNN was applied to concentration changes. The training dataset was obtained through a semi-simulation process, and the size of each package was fixed as 1,024 samples. If the fNIRS data are obtained at a low sampling frequency, the package size is sufficient, but the filtering performance for high-sampling-frequency fNIRS data remains unknown. The matrix structure of the input data enables an easy adaptation to fNIRS measurement with multi-distance configurations. However, similar to the GLM-based method, the information of the stimulation paradigm is a prerequisite in the input. The inclusion of the stimulation paradigm is critical to obtain good performance of the method.

### Comparison of diverse solutions

The methods presented above are the fundamental techniques available from the existing MA removal solutions. Among the new techniques under investigation, many solutions were developed from or in stack of one or several basic techniques. Table 5 demonstrates the relationship between other new solutions and these basic techniques. For a fair comparison among these techniques, systematic approaches are required and the results are presented.

**TABLE 5:** List of motion artifact (MA) removal approaches and their relationship.

Paper	Solution name or category	Validation data type	Age group	Motion artifact type	Input	Source code availability
Izzetoglu et al. (2005)	Wiener filtering	Experimental	Adults		Optical intensities	Matlab
Zhang et al. (2005)	principal component analysis (PCA	Experimental	Adults		Concentration changes	Homer3
Lee et al. (2007)	Periodic moving average	Experimental	Adults		PPG signals	No
Blasi et al. (2010)	Channel rejection, adaptive filtering	Experimental	Infants		Concentration changes	Matlab
Cui et al. (2010)	Correlation-based signal improvement (CBSI)	Experimental	Adults	Spike	Concentration changes	Homer3
Izzetoglu et al. (2010)	Discrete Kalman filter	Experimental	Adults		Optical intensities	Matlab
Scholkmann et al. (2010)	Moving standard deviation, movement artifact reduction algorithm (MARA)	Experimental	Adults	Spike + drift	Optical densities	Homer3
Kim et al. (2011)	Active noise cancellation (ANC)	Experimental	Adults	Spike	Optical intensities	No
Virtanen et al. (2011)	Accelerometer-based motion artifact removal (ABAMAR)	Experimental	Adults	Spike + drift	Optical intensities and accelerations	No
Molavi and Dumont (2012)	Wavelet-based method	Experiment	Infants	Spike	Optical densities	Homer3
Amian and Setarehdan (2013)	ARMA model-based KF	Simulated + experimental	Adults		Optical intensities	No
Barker et al. (2013)	AR model and reweighted least squares	Simulated + experimental	Infants	Spike + drift	Optical intensities	No
Santosa et al. (2013)	Independent component analysis (ICA)	Experimental	Adults		Concentration changes	MNE-Python
Sweeney et al. (2013)	Ensemble empirical mode decomposition with canonical correlation analysis (EEMD-CCA)	Simulated + experimental	Adults		Optical intensities	No
Takahashi et al. (2013)	Motion artifact reconstruction	Experimental	Adults		Concentration changes	No
Selesnick et al. (2014)	Transient artifact reduction algorithm (TARA)	Simulated + experimental	Adults	Spike + drift	Optical intensities	https://ieeexplore.ieee.org/abstract/document/6942269/media#media
Yucel et al. (2014b)	Collodion-fixed prism-based optical fibers	Experimental	Adults	Spike + drift	Optical intensities	No
Yucel et al. (2014a)	Targeted PCA	Experimental	Adults	Spike + drift + oscillation	Optical intensities	Homer3
Chiarelli et al. (2015)	Kurtosis-based wavelet filtering (kbWF)	Semi-simulated	Adults	Spike	Optical densities	No
Metz et al. (2015)	MARA + ABAMAR	Experimental	Adolescents	Spike + drift	Optical densities + acceleration	No
Yamada et al. (2015)	Multidistance optode arrangement technique	Experimental	Phantom	Spike + drift	Optical intensities	No
Barker et al. (2016)	Kalman autoregressive, iterative robust least-squares functional near-infrared spectroscopy (fNIRS) model, KF	Semi-simulated	Adults	Spike	Optical intensities + stimulated function	AnalyzIR
Gu et al. (2016)	Empirical mode decomposition (EMD) + MARA	Semi-simulated	Children	Spike + drift	Optical intensities	No
Guerrero-Mosquera et al. (2016)	CBSI-based automatic artifact detection	Experimental	Adults		Concentration changes	No
Islam et al. (2017)	Multi-stage cascaded adaptive filtering (RLS) + singular spectrum analysis (SSA)	Experimental	Adults		Optical intensities + acceleration	No
Santosa et al. (2017)	Robust correlation of the innovations models	Simulated + experimental	Children		Optical intensities	www.bitbucket.org/huppertt/
Dong and Jeong (2018)	EKF with non-linear state-space model and short separation	Semi-simulated	Adults		Concentration changes	No
Jahani et al. (2018)	MARA, Savitzky-Golay filtering	Semi-simulated + experimental	Adults	Spike + drift	Optical densities	Homer3
Lee et al. (2018)	Wavelet-decomposed back-propagation neural network (BPNN) + contaminated channel identification algorithm based on entropy	Experimental	Adults	Spike + drift	Optical intensities	No
Nguyen et al. (2018)	Adaptive filtering based on RLS with an exponential forgetting factor	Simulated + experimental	Adults	Spike	Concentration changes	No
Shukla et al. (2018)	Stationary wavelet transforms, zero-mean Laplace distribution modeling	Experimental	Adults	Spike	Original EDA signals	No
Siddiquee et al. (2018)	Autoregression with exogenous input	Experimental	Adults		Concentration changes	No
Sutoko et al. (2018)	Adaptive algorithm, rejection	Simulated + experimental	Children		Product of concentration changes and optical path length	No
Fishburn et al. (2019)	TDDR + robust regression	Simulated + experimental	Children	Spike + drift + oscillation	Optical densities	MNE-Python
Seghouane and Ferrari (2019)	Robust fNIRS HRF estimation algorithm	Simulated + experimental	Adults		Optical intensities	No
von Luhmann et al. (2019)	Blind source separation and accelerometer-based artifact rejection and detection (BLISSA2RD), ICA + Canonical Correlation Analysis and temporal embedding	Simulated + experimental	Adults		Optical intensities	https://github.com/avolu/BLISSARD
Arunkumar and Bhaskar (2020)	Cascaded RLS, normalized least mean square (NLMS), LMS adaptive filter	Experimental	Adults		Optical intensities + acceleration	No
Sherafati et al. (2020)	Global variance of temporal derivatives (GVTD)	Experimental	Adults + infants		Optical densities or concentration changes	No
Zhao J. et al. (2020)	Targeted median filter and mathematical morphology (tMedMor)	Semi-simulated	Adults	Spike + drift + oscillation	Optical densities	No
Lacerenza et al. (2021)	TD-fNIRS	Experimental	Adults		Photon distribution of time-of-flight	No
Perpetuini et al. (2021)	Wavelet-based method, wavelet coherence (WCOH), video tracking	Semi-simulated	Adults	Spike + oscillation	Optical densities	No
Zhou et al. (2021)	NIRS-ICA toolbox, PCA	Simulated + experimental	Adults		Optical intensities	NIRS-ICA
Gao L. et al. (2022)	MARA + wavelet	Semi-simulated	Adults	Spike + drift + oscillation	Concentration changes	Homer3
Kim et al. (2022)	U-net CNN	Semi-simulated	Adults	Spike + drift + oscillation	Concentration changes	No
Huang et al. (2022)	Dual-stage median filter	Simulated + experimental	Adults	Spike + drift	Optical intensities	https://gitee.com/cognoholic/differential-median-filter.git
Gao Y. et al. (2022)	Deep learning-based CNN	Simulated + experimental	Adults	Spike + drift + oscillation	Concentration changes	https://github.com/YuanyuanGao216/fNIRS_denoise_by_DL

Comparisons have been made among the trial rejection method, recursive least squares adaptive filtering method (Park et al., 2020), wavelet-based method, ICA, two- or multi-channel regression method, Kalman filter, MARA, PCA, moving average, band-pass filter, median filter, Savitzky–Golay filter, and CBSI for adults’ fNIRS data. Overall, the MA correction approach is better than the trial rejection approach (Brigadoi et al., 2014). MA correction can also help to improve classification accuracy (Janani and Sasikala, 2017). Wavelet-based method, ICA, multiple-channel regression, and MARA had a good performance in noise suppression. When processing children’s fNIRS signals, researchers compared PCA, MARA, wavelet-based method, moving average, CBSI, and tPCA. MARA, tPCA, and CBSI can retain a higher number of trials. The tPCA and MARA are more robust. Moving average, wavelet-based method, and tPCA have a better performance than other methods. Researchers also examined the filtering performance of infants’ fNIRS data. Trial rejection, wavelet-based method, tPCA, MARA, and different processing pipelines were compared in their studies. MA correction can retain many trials, but hemodynamic response functions were also suppressed. The optimal pre-processing pipeline depends on the dataset (Gemignani and Gervain, 2021). Similar to the conclusion for adults’ data, MA correction is better than trial rejection. The wavelet-based method alone or in a stack with MARA and the wavelet-based method (iqr = 0.5) had better performance in noise suppression. MARA in a stack with the wavelet-based method can recover a higher number of trials.

Reportedly, the wavelet-based filter has good performance when MAs are caused by sudden large movements (or spikes). Moreover, ICA and multi-channel regression also perform well in terms of noise suppression (Robertson et al., 2010). Cooper et al. (2012) reported that PCA, MARA, wavelet-based method, Kalman filter, and trial rejection method could reduce the MSE in corrupted fNIRS signals (Cooper et al., 2012). PCA, MARA, and wavelet-based methods exhibited much better performance than the other methods (Piper et al., 2014). In contrast, Brigadoi et al. (2014) claimed that the Kalman filter performed better using the area under the curve (AUC ratio) of the mean hemodynamic responses than PCA, and MARA, followed by the wavelet-based method and CBSI. They also claimed that the wavelet-based method exhibited a better performance in reducing the AUC for the first two seconds after stimulus onset (AUC_0–2_).

**FIGURE 5:**
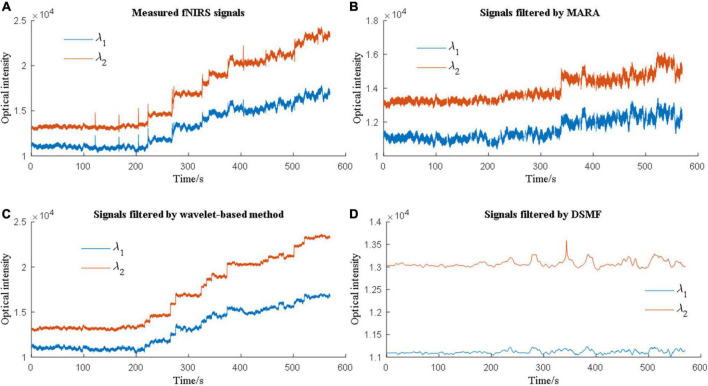
Different motion artifact (MA) removal techniques have different performances when processing experimental data.

Therefore, the evaluation of MA removal techniques depends on the evaluation metrics of the researchers. Due to this phenomenon, multiple metrics need to be considered when comparing various methods. Cooper et al. (2012) used MSE to assess how different solutions can suppress MAs and contrast-to-noise ratio (CNR) to evaluate how well the solutions can retain brain activation signals. Janani and Sasikala (2017) reported that a band-pass filter, median filter, and Savitzky-Golay filter could increase the CNR. Additionally, the performance of the MA solutions differs from each other when the MA types are different. Figure 5 showed an example of MA removal using different filters.

The optimal solution for different age groups can be different. Hu et al. (2015) tested the MA removal performance of six solutions using fNIRS data from children: wavelet-based method, PCA, MARA, moving average, CBSI, and the combination of moving average and wavelet-based methods. It was found that MARA successfully suppressed all types of MAs but had an unsatisfactory AUC ratio. Moving average and wavelet-based method turned out to be better solutions for children. Meanwhile, CBSI was efficient in removing spike-like and fast step-like MAs. Besides, Reyes et al. (2018) claimed that tPCA was the best solution for processing children’s fNIRS signals and the authors added that CBSI occasionally produced unstable hemodynamic responses. However, the authors also claimed that MARA was robust and performed well in both AUC and the average standard deviation of each trial-specific hemodynamic response. The contradiction between Reyes and Hu’s findings regarding the MARA originates from improper artifact correction (Hu et al., 2015). Another study by Di Lorenzo et al. (2019) used a combination of MARA and wavelet-based methods and found it to be more effective for infant data than using trial rejection, MARA, or wavelet alone. They further justified that using MA correction could retain many trials. Similar results were obtained by Behrendt et al. (2018). They claimed that trial rejection was not recommended for infants’ data. Table 6 summarizes the data type, age group, techniques in comparison, and main conclusions of all nine comparison papers.

**TABLE 6:** Data type, age group, motion artifact (MA) removal techniques and main conclusions in the studies of MA comparison.

Paper	Data type	Age group	Techniques of interest	Main conclusions
Robertson et al. (2010)	Experimental	Adults	(i) RLS adaptive filtering (ii) Wavelet-based method (iii) ICA (iv) Two-channel regression (v) Multi-channel regression	(i) Independent component analysis (ICA) or multiple-channel regression has the largest SNR changes. (ii) RLS adaptive filtering produced the smallest SNR improvement. (iii) Wavelet-based method is most effective for spike-like artifacts. (iv) ICA and multiple-channel regression are good options when time-consuming manual techniques are absent.
Cooper et al. (2012)	Semi-simulated	Adults	(i) PCA (ii) Movement artifact reduction algorithm (MARA) (iii) Wavelet-based method (iv) Kalman filter	(i) All methods yield a significant reduction in mean square error (MSE) and an increase in contrast-to-noise ratio (CNR). (ii) MARA has the largest drop in MSE. (iii) Wavelet-based method has the highest average increase in CNR. (iv) The authors recommend the routine application of MA correction.
Brigadoi et al. (2014)	Experimental	Adults	(i) Trial rejection (ii) PCA (iii) MARA (iv) Wavelet-based method (v) Kalman filter (vi) Correlation-based signal improvement (CBSI)	(i) MA correction is better than trial rejection. (ii) Wavelet-based method is the most powerful method.
Hu et al. (2015)	Experimental	Children	(i) PCA (ii) MARA (iii) Wavelet-based method (iv) Moving average (v) CBSI (vi) Wavelet + moving average	(i) Moving average and wavelet-based method outstand the others. (ii) Types C and D artifacts have a larger influence on GLM model than Types A and B*.
Janani and Sasikala (2017)	Experimental	Adults	(i) Band-pass filter (ii) CBSI (iii) Median filter (iv) Savitzky–Golay filter (v) Wavelet denoising, ICA	(i) Wavelet-based method attenuates the MA energy and increases the CNR of Subjects 1 and 2. (ii) ICA also suppresses physiological noises and spike-like MAs. (iii) MA removal helps to classify motor tasks.
Behrendt et al. (2018)	Semi-simulated	Infants	(i) Trial rejection (ii) Wavelet-based method (iii) Targeted PCA (tPCA) (iv) tPCA + wavelet-based method	(i) The authors suggested using the wavelet-based method with iqr = 0.5. (ii) Trial rejection alone is not recommended. (iii) Assessing MA removal performance may not require full simulation studies.
Reyes et al. (2018)	Experimental	Children	(i) tPCA (ii) MARA (iii) Wavelet-based method (iv) CBSI	(i) tPCA, MARA, and CBSI retained a higher number of trials. (ii) The CBSI produced sometimes unstable hemodynamic response functions. (iii) The tPCA and MARA were the most robust in all metrics. (iv) The tPCA outperformed MARA. (v) The tPCA is an effective technique in MA correction for young children.
Di Lorenzo et al. (2019)	Semi-simulated + experimental	Infants	(i) Trial rejection (ii) MARA (iii) Wavelet-based method (iv) MARA + wavelet-based method	(i) MA correction is better than trial rejection. (ii) Wavelet-based method alone or in stack with MARA are the most effective in reducing the between- and within-subject standard deviation. (iii) MARA + wavelet-based method performs the best in semi-simulation and recovering the greatest number of trials.
Gemignani and Gervain (2021)	Simulated + experimental	Infants	Five pipelines	(i) MA correction can retain many trials. (ii) Hemodynamic response functions were suppressed as well. (iii) MA rejection preserved adequately the characteristics of HRF. (iv) The performance of all pipelines declined when noise power increased but less than no pre-processing. (v) No difference in running pre-processing on either optical densities or concentration changes. (vi) Pre-processing depends on the dataset.

*Type A: Spike-like MA with a standard deviation (SD) of 50 from the mean within 1 s. Type B: Spike-like MA with a SD of 100 from the mean within 1 to 5 s. Type C: Baseline shift with a gentle slope between 5 and 30 s with a SD of 300 from the mean. Type D: Slow baseline shift longer than 30 s with a SD of 500 from the mean.

## Evaluation metrics

Various evaluation metrics have been defined in the literature for noise suppression and signal distortion to quantitatively evaluate the performance of artifact removal solutions. Some metrics have been defined to describe the extent to which the solutions could suppress the artifacts, while others were designed to assess the signal distortion after data processing. In this section, we introduce the details of several metrics for artifact removal except for some commonly used metrics such as root-mean-squared error (to assess the square-root distance between filtered signals and reference signals), the area under the curves (AUC. e.g., researchers can take the initial 1–2 s and the 2–6 s of HbO concentrations) (Reyes et al., 2018), and the *t*-test (e.g., used together with GLM) (Hu et al., 2015).

### Noise suppression

#### Signal to noise ratio

The signal-to-noise ratio (ΔSNR) in motion artifact removal (Izzetoglu et al., 2005) is different from the more commonly known signal-to-noise ratio (SNR), which is simply the ratio between the power of real signals and the power of noises expressed in decibel. ΔSNR (Δ*SNR*) is defined as the difference between the estimation SNR (*SNR*_*e*_) and the input SNR (*SNR*_*i*_). The two variables are defined as follows.


(87) $$SNR_{e}=10{\text{log}}_{10}\left(\frac{\text{Var}\left(x\right)}{\text{Var}\left(\hat{x}-x\right)}\right)$$

(88) $$SNR_{i}=10{\text{log}}_{10}\left(\frac{\text{Var}\left(x\right)}{\text{Var}\left(z-x\right)}\right)$$

The operator Var(⋅) denotes the variance of the variable in the parentheses. A hat over the motionless fNIRS signal *x* denotes the estimated motionless fNIRS signal, and *z* denotes the measured fNIRS signal. Subsequently, Δ*SNR* is defined as follows.


(89) $$\Delta SNR=SNR_{e}-SNR_{i}$$

If the filter of interest can perfectly remove motion artifacts in *z*, then Δ*SNR*→ + ∞. If Δ*SNR* < 0, the filter of interest amplifies the artifacts in the measured signal. Otherwise, the filter can partially remove motion artifacts. In contrast, the larger the ΔSNR, the better the performance of the filter. Physically, the metric describes how well the filter can attenuate motion artifacts in the measured signals.

Since a motionless fNIRS signal is required in the calculation, ΔSNR can be well suited to evaluate the filtering performance of the simulated data. When applied to experimental data, a desired motionless signal is required in place of the motionless fNIRS signal.

#### Contrast to noise ratio

The contrast to noise ratio (CNR) describes the difference in the fNIRS signal before and during stimulation (Zhang et al., 2005). Here, the subscript “pre” denotes any signal before the stimulus, and the subscript “dur” denotes any signal during the stimulus. The CNR can then be computed as follows.


(90) $$CNR=\frac{{\bar{\hat{x}}}_{\text{dur}}-{\bar{\hat{x}}}_{\text{pre}}}{\sqrt{\sigma_{\text{dur}}^{2}+\sigma_{\text{pre}}^{2}}+\epsilon }$$

where σ_*dur*_ and σ_*pre*_ denote the standard deviations of the filtered fNIRS signals during and before the stimulus, and *ϵ* corresponds to a small non-negative constant compensating for channels with a small stimulus response but relatively low activity. Empirically, ∈= 0.1 μM. In some studies, ∈ was ignored. When MAs exist, the CNR map may appear as all active or negative channels. The active and inactive areas spread throughout the map. Once MAs are properly removed, CNR maps with more concise active zones can be obtained (Kohno et al., 2007).

#### Percent root difference

The percent root difference (PRD) is a metric that describes how well the two signals match each other (Scholkmann et al., 2010; Dong and Jeong, 2018). When using the motionless signal *x* as a reference, the PRD is defined as follows.


(91) $$PRD=100\%\times \sqrt{\sum_{i=1}^{N}\frac{{\left(\hat{x}\left(i\right)-x\left(i\right)\right)}^{2}}{\sum_{i=1}^{N}x^{2}\left(i\right)}}$$

The smaller the PRD, the better the filtered signals match the motionless signals, that is, fewer artifacts exist in the filtered signals.

#### Artifact power attenuation

Artifact power attenuation (APA) indicates how much the artifacts in the measured signal can be suppressed in the signal of interest within the corrupted segments (Molavi and Dumont, 2012). Therefore, the metric is expressed in decibels as follows.


(92) $$APA=10\log_{10}\frac{\sum_{i\in A_{m}}{\left(z^{\text{HP}}\left(i\right)\right)}^{2}}{\sum_{i\in A_{m}}{\left({\hat{x}}^{\text{HP}}\left(i\right)\right)}^{2}}$$

where *A*_*m*_ denotes the set of time segments in which the MAs occur. The set constrains the evaluation domain within corrupted segments. The superscript HP denotes a high-pass signal. It is introduced to remove low-frequency physiological variants (Molavi and Dumont, 2012). Since the measured signals are placed in the nominator, the larger the APA, the better the filter of interest that can suppress MAs. Typically, APA is expressed in decibels (Shukla et al., 2018).

APA can directly reveal a filter’s resistance to MAs without disturbance from physiological noise. When using APA, the high-pass filter needs to be chosen carefully to ensure efficient filtering of the physiological noise. Importantly, the filters for both the nominator and denominator should be identical to facilitate a fair comparison.

#### Within/between-subject standard deviation

Within-subject standard deviation and between-subject standard deviation are two different metrics for evaluating the performance of the MA filter on the experimental data. Within-subject standard deviation refers to the average standard deviation of single-trial hemodynamic responses for a given channel and chromophore. *N*_*trl*_ denotes the number of trials in the signals, *k* denotes the sample index, *i* denotes the trial index, and *N* denotes the number of samples in a trial. Δ$\hat{c}$_*i*_(*k*) is the estimated concentration change at instance *k* in trial *i*.


(93) $$\sigma_{\text{within}}=\frac{\sum_{k=1}^{N}{\text{std}}_{i}\left(\Delta {\hat{c}}_{i}\left(k\right)\right)}{N},i=1,\text{…}\;,N_{\text{trl}}$$

In practice, there is a within-subject standard deviation for each chromophore in each channel of each subject. The metric is used under the assumption that most of the variability between single-trial hemodynamic responses for each subject is because of the influence of MAs. When comparing the two signals, the within-subject standard deviations can be computed for each chromophore, channel, and subject. Then, the two values were mapped to a point in a scatter plot. We can compare the filtering performance of the two filters by counting the number of points below or above the line *y* = *x*.

The between-subject standard deviation refers to the standard deviation of the averaged hemodynamic responses across subjects for each channel (Brigadoi et al., 2014; Di Lorenzo et al., 2019). *N*_*sub*_ denotes the number of subjects, *i* denotes the subject number, *N* denotes the number of samples in an experiment, and Δ$\hat{c}$_*i*_(*k*) denotes the estimated concentration change at instant *k* of subject *i*. The between-subject standard deviation becomes


(94) $$\sigma_{\text{between}}=\frac{\sum_{k=1}^{N_{\text{sub}}}{\text{std}}_{i}\left(\Delta {\hat{c}}_{i}\left(k\right)\right)}{N},i=1,\text{…}\;,N_{\text{sub}}$$

It is assumed that the MAs dominate the variability of hemodynamic responses across subjects when using the metric.

### Signal distortion

#### Correlation coefficient

There are two methods for evaluating the motion artifact removal performance using the correlation coefficient. The first method computes the correlation between a reference signal and a block-average signal (Zhang et al., 2005). The reference signal was obtained by shifting the rectangular pulse signal to the right. The desired metric is then calculated as the correlation coefficient between the reference signal and the block-averaged response of the filtered signal.

The second method defines the difference in the correlation coefficient (ΔCC) (Izzetoglu et al., 2005; Jahani et al., 2018) as the difference between the estimated correlation coefficient and the input correlation coefficient, that is,


(95) $$\Delta CC=\text{corr}\left(x,\hat{x}\right)-\text{corr}\left(x,z-x\right)$$

The operator corr(⋅, ⋅) represents the correlation between two variables in brackets. The second term in this definition tends to be zero. If ΔCC < 0, then the filter of interest significantly distorts the motionless signal. As ΔCC is closer to one, the distortion reduces in the filtered signal.

Similar to ΔSNR, ΔCC is well-suited for simulated data. When applied to experimental data, reference motionless signals are required in place of motionless fNIRS signals. In contrast, the first definition of the correlation metric requires an experimental paradigm, primarily because it compares the shifted rectangular pulse signal and the block-averaged response.

#### Pearson product-moment correlation coefficient

This metric appeared in 2008 and was designed to measure the similarity between two signals (Scholkmann et al., 2010). The coefficient is defined as follows.


(96) $$r=\frac{1}{M}\sum_{i=1}^{N}\left(\frac{\hat{x}\left(i\right)-\bar{\hat{x}}\left(i\right)}{\sigma_{\hat{x}}}\right)\left(\frac{x\left(i\right)-\bar{x}\left(i\right)}{\sigma_{x}}\right)$$

where σ denotes the standard deviation and *M* = *N*–1. Moreover, the coefficient can also be used to evaluate the similarity between the motionless signal *x* and measured signal *z*. The only difference is the replacement of the estimated motionless signal with *z* in the equation. It is used to calculate the improvement of the proposed method compared with the measurement. The more significant the absolute difference between *r*($\hat{x}$, *x*) and *r*(*x, z*), the better the performance of the proposed method. As *r* becomes closer to one, there is less distortion in the signal of interest. Occasionally, the difference in the Pearson product-moment correlation coefficient can also be used to evaluate the improvement of the filtered signals. The difference is defined as follows.


(97) $$\Delta r=r\left(\hat{x},x\right)-r\left(z,x\right)$$

The difference may be expressed as a percentage.

### Data type compatibility

According to Table 5, three types of data exist: (i) Simulated data, (ii) semi-simulated data, and (iii) experimental data. A semi-simulated dataset can be generated by adding desired concentration changes to MA-corrupted experimental data at resting state, or by adding uncorrupted experimental data at the resting state to the sum of desired concentration changes and designed artifact sequence. For both simulated and semi-simulated datasets, the ground-truth motionless signals are known. Nevertheless, this is not true for experimental datasets. Therefore, the input requirements of each metric decide the metric’s compatible data types. Table 7 summarizes the data type compatibility of different metrics.

**TABLE 7:** Suitability checking of the defined metrics to three types of data.

Metrics	Evaluation function	Simulated data	Semi-simulated data	Experimental data
ΔSNR	Noise suppression	√	√	
CNR	Noise suppression	√	√	√
PRD	Noise suppression	√	√	
APA	Noise suppression	√	√	√
Within-/between-subject SD	Noise suppression	√	√	√
ΔCC	Signal distortion	√	√	
Pearson product-moment correlation coefficient	Signal distortion	√	√	

## Conclusion and outlook

This review suggests that removing MAs is unavoidable in developing a more generalized fNIRS device. Literature reported that fNIRS demonstrate a good tolerance to motion artifacts when well-positioned (Pinti et al., 2020). However, a generalized fNIRS apparatus may not guarantee that all users will stick to this rule. The introduction of motion artifact removal techniques will add to the device’s robustness and stability even when the users are involved in significant physical activities. These improvements will add to a positive user experience. The MA issue will become crucial for portable and wearable fNIRS devices. Once the problem is solved, we can envision a more exciting application of fNIRS to connect people or connect the real world with the digital world. Additionally, Tables 5 and 6 show that the filtering performance of one method on different age group can be divers. Hence, for a specific application, a study regarding the optimal solution is still necessary. Our in-depth review can help experienced researchers as well as novices in three ways. First, a brief introduction to some core solutions from a different perspective can help gain a better understanding as to how the solutions can be replicated, whether they are suitable for online or offline applications, and their advantages and disadvantages. Second, the definitions and physical meanings of commonly used metrics were summarized. Researchers can select the best metrics according to their requirements. Third, a sketch of the latest developments in MA removal techniques can help readers familiarize themselves with the field and decide their research focus. This review can be served as a list of different motion artifact removal solutions. When targeting the application of fNIRS, readers can pick the methods or metrics fulfilling their requirements for their specific scenario. When attacking a better noise removal solution, readers can develop their theory from the methods mentioned in this work or take another path.

The research in MA removal has made significant progress since the early 21st century. Different attempts have been made to remove MAs, especially for offline processing. Some researchers have also investigated the role of MA removal techniques in fNIRS data analysis pipelines (Gemignani and Gervain, 2021). Some studies managed to find out the optimal techniques among several candidates when doing experiments on a targeted population (Hu et al., 2015; Behrendt et al., 2018; Reyes et al., 2018; Di Lorenzo et al., 2019). Some attempted to solve the problem by improving the optical models (Scholkmann et al., 2014b; Yamada et al., 2015).

However, this review also revealed several lacunae concerning MA removal. First, no research has explained how to achieve a balance between the use of auxiliary hardware and that of an algorithmic solution. A comparative study compared an adaptive filter (accelerometer is needed) with wavelet-based filtering, ICA, two-channel, and multi-channel regression (Robertson et al., 2010); however, it is far from sufficient. Second, the existing online MA removal solutions are primarily validated for noise suppression and signal distortion, but their filtering delays are not evaluated. An online solution with high noise suppression, low signal distortion, and low delay is significant for portable and wearable fNIRS devices. Third, the robustness and compatibility of the solutions in different application scenarios were not thoroughly studied. A solution’s filtering performance may change when the user’s age, tasks, external disturbance, or user’s physical health are different. This issue is crucial for developing application-oriented fNIRS devices, such as devices that operate in extreme environments or a targeted population.

The research to remove MAs is not a method-oriented topic but an application-oriented topic. Any solution that can help make one step further to either general applications or a specific application would be advantageous to the fNIRS community.

## Author Contributions

RH and K-SH designed the structure and scope of the review. RH aggregated reviewed articles and prepared the first draft manuscript. DY and GH verified the equations. K-SH reviewed and revised the manuscript. All authors contributed to the article and approved the submitted version.
